# Nomogram Predicting the Likelihood of Parametrial Involvement in Early-Stage Cervical Cancer: Avoiding Unjustified Radical Hysterectomies

**DOI:** 10.3390/jcm9072121

**Published:** 2020-07-05

**Authors:** Louise Benoit, Vincent Balaya, Benedetta Guani, Arnaud Bresset, Laurent Magaud, Helene Bonsang-Kitzis, Charlotte Ngô, Patrice Mathevet, Fabrice Lécuru

**Affiliations:** 1 Faculty of Medicine, Paris University, 75006 Paris, France; vbalaya@hotmail.com (V.B.); fabric.lecuru@curie.fr (F.L.); 2 Gynecology Department, Centre Hospitalo-Universitaire Vaudois, 1011 Lausanne, Switzerland; benedetta.guani@hotmail.it (B.G.); patrice.mathevet@chuv.ch (P.M.); 3 University of Lausanne, Department of Gynecology and Obstetrics, 1011 Lausanne, Switzerland; 4 Gynecology and Obstetrics Department, Beaujon Hospital, 92110 Clichy, France; abresset@gmail.com; 5 Public Health Department, Hospices Civils de Lyon, 69002 Lyon, France; Laurent.magaud@chu-lyon.fr; 6 Gynecological and Breast Surgery and Cancerology Center, RAMSAY-Générale de Santé, Hôpital Privé des Peupliers, 75013 Paris, France; h.kitzisbonsang@yahoo.fr (H.B.-K.); charlotte.ngo2@gmail.com (C.N.); 7 Breast, Gynecology and Reconstructive Surgery Unit, Curie Institute, 75005 Paris, France

**Keywords:** cervical cancer, nomogram, parametrium, parametrial involvement, radical hysterectomy, radical trachelectomy

## Abstract

Background: We aimed to establish a tool predicting parametrial involvement (PI) in patients with early-stage cervical cancer and select a sub-group of patients who would most benefit from a less radical surgery. Methods: We retrospectively reviewed patients from two prospective multicentric databases—SENTICOL I and II—from 2005 to 2012. Patients with early-stage cervical cancer (FIGO 2018 IA with lympho-vascular involvement to IIA1), undergoing radical surgery (hysterectomy or trachelectomy) with bilateral sentinel lymph node (SLN) mapping with no metastatic node or PI on pre-operative imaging, were included. Results: In total, 5.2% patients (11/211) presented a histologic PI. After univariate analysis, SLN status, lympho-vascular space invasion, deep stromal invasion and tumor size were significantly associated with PI and were included in our nomogram. Our predictive model had an AUC of 0.92 (IC95% = 0.86–0.98) and presented a good calibration. A low risk group, defined according to the optimal sensitivity and specificity, presented a predicted probability of PI of 2%. Conclusion: Patients could benefit from a two-step approach. Final surgery (i.e. radical surgery and/or lymphadenectomy) would depend on the SLN status and the probability PI calculated after an initial conization with bilateral SLN mapping.

## 1. Introduction

Radical hysterectomy with pelvic node assessment is the standard treatment in early-stage cervical cancer. Indeed, parametrial involvement (PI) is reported in 4–20% of these patients [1,2,3] and constitutes a key prognostic factor in these patients [4]. 

Autonomic nerves, consisting in sympathetic fibers (hypogastric nerve) and parasympathetic fibers (pelvis splanchnic nerve and inferior hypogastric plexus), cross this rich lymph-vascular space [5,6]. Therefore, radical hysterectomy presents a high surgical morbidity due to extensive ureteral and nerve dissection [7]. They may be responsible for post-operative complications, such as bladder or bowel dysfunction, urinary tract fistula and urinary tract injury [8,9]. However, the resection of the parametrium is of primary importance in early stage cervical cancer, since it may be the main site of disease recurrences [10].

In this specific setting, decreasing the morbidity associated with a radical surgery without jeopardizing survival outcomes after parametrectomy is of paramount importance. One option to decrease the morbidity linked to radical surgery is nerve-sparing surgery [11]. Several techniques of nerve-sparing surgery have been described [7,11,12,13]. However, these techniques involve specific learning curves and require high surgical skills before being generalized. Likewise, Querleu and Morrow suggested a classification defining different types of radical hysterectomies according to the extent of parametrial resection [14]. Patients can, therefore, benefit from a tailored surgery, where the radicality is based on prognostic factors [15,16].

It has been shown that a subpopulation of patients presents a low risk of PI. Several studies have described variables associated with PI: age, tumor size, body-mass index, menopausal status, lymph-vascular space involvement (LVSI) and deep stromal invasion (DSI) [1,2,3,4,17,18,19,20,21]. As an alternative to nerve-sparing radical hysterectomy, another strategy would be to accurately select these patients who would not benefit from a parametrectomy. One must balance the survival benefit of such a radical hysterectomy with the risks it imposes. In a large retrospective study, Covens et al. stated that patients with a tumor size < 20 mm, no LVSI, a DSI < 10 mm and negative pelvic node had a probability of PI of 0.6% and could, therefore, benefit from less radical surgery [1]. Magnetic resonance imagery (MRI) could also help define parametrial involvement [22,23]. However, in a systematic review, it was found that MRI had a sensitivity of 74% in predicting PI [24]. Even fusion imaging can only predict PI in 80–86% of the cases [25].

In this study, we aimed to develop a simple tool to predict parametrial involvement in early-stage cervical cancer and define a low-risk group of patients who would least benefit from a parametrectomy by integrating simple clinicopathologic factors and SLN status.

## 2. Material and Methods

### 2.1. Population and Data Analysis

We retrospectively reviewed the results from two prospective multicentric studies evaluating the benefit of sentinel lymph node in cervical cancer—SENTICOL I and II. Patients with early-stage cervical cancer (stage IA with lympho-vascular involvement to stage IIA1) were included from seven French gynecological oncology centers between 2005 and 2007 and from 23 centers from 2009 to 2012 for SENTICOL I and II, respectively. The Paris Descartes Ethical Committee (Comité de Protection des Personnes HEGP-Broussais) approved this study. The patients included in the two studies gave a written consent stating the use of data for secondary analyses.

We included patients undergoing radical surgery (radical hysterectomy or trachelectomy) with bilateral sentinel lymph node mapping for early stage cervical cancer with no metastatic node or PI on pre-operative imaging. Patients without radical surgery were excluded since PI was not assessable. Likewise, patients with a clinical or MRI PI were not included. Patients with a pre-operative brachytherapy were also excluded. Patients with early stage cervical cancer from FIGO IA1 with LVSI to FIGO IIA1 were analyzed. We chose to include patients with FIGO IA1 with poor prognosis (LVSI) and FIGO IA2, since no clear recommendations exist on the importance of a parametrectomy in these patients (grade C ESMO/ESGO recommendations).

Data were collected regarding: (i) demographic characteristics (age, body-mass index); (ii) surgery (type of surgery, sentinel lymph node, conization); (iii) tumor characteristics (FIGO stage, histological examination, imagery). On the conization specimen information, regarding tumor size, histology type, presence or not of LVSI, DSI and margin status were available. The ideal threshold of DSI predicting PI was defined using a receiver operating characteristics (ROC) curve. 

All specimens were analyzed by experienced gynecologic pathologists at each center. PI was defined as the presence of the disease in the parametrial tissue. The sentinel lymph node (SLN) was detected by a combined labeling technique (Radioactive tracer [99 mTc] and patent Blue). SLNs were examined after hematoxylin and eosin (HE) staining of 200 µm sections. SLNs, defined as negative by HE, were then analyzed by immunohistochemistry with anti-cytokeratin AE1–AE3 antibodies. Isolated tumor cells (ITCs) were defined as <0.2 mm, micrometastases as between 0.2 and 2 mm, and macrometastases as >2 mm. 

### 2.2. Statistical Analysis

Patients were separated into two groups according to their PI at the final pathological exam. A Student-t test and chi^2^ test were used to compare the continuous and categorical values, respectively. A two-tailored *p*-value of 0.05 was considered significant. 

### 2.3. Development of the Model

We developed a nomogram to predict patient-specific likelihoods of PI. Backward variable selection was performed to determine independent predictors. Values for each of the model covariates were mapped to points on a scale ranging from 0 to 100. The total points obtained for each model corresponded to the probability of a PI.

### 2.4. Accuracy of the Model

The predictive accuracy of the model was assessed by its discrimination and calibration [26] Discrimination was assessed using to the area under the curve (AUC) on the ROC curve. The AUC is a measure of the ROC that reflects the ability of a test to discriminate the outcomes across all possible levels of positivity. AUC ranges from 0 to 1 and a model is considered to have a poor, fair or good performance if the AUC lies between 0.5 and 0.6, 0.6 and 0.7 or is greater than 0.8, respectively [27].

Calibration was assessed using plots that overlap the prediction model. Average and maximal errors between predictions and observations obtained from the calibration curve were estimated.

### 2.5. Validation of the Model

An internal validation (with 200 bootstrap resamples to obtain relatively unbiased estimates) was also performed. The bootstrapping method is based on resampling obtained by randomly drawing data and replacing them with samples from the original dataset. It provides an estimate of the average optimism of the AUC [26]. No external validation was carried out.

### 2.6. Optimal Threshold of the Model

The optimal threshold of our model, classifying patients as low risk or high risk of PI, was defined using the Youden index [28]. At this threshold, the sensitivity, specificity, negative predictive values (NPVs) and positive predictive values (PPVs) were assessed. 

All statistical analyses were carried out using an Excel database and Rstudio, version 1.1.447 (https://www.r-project.org/).

## 3. Results

### 3.1. Population Characteristics

**Figure 1:**
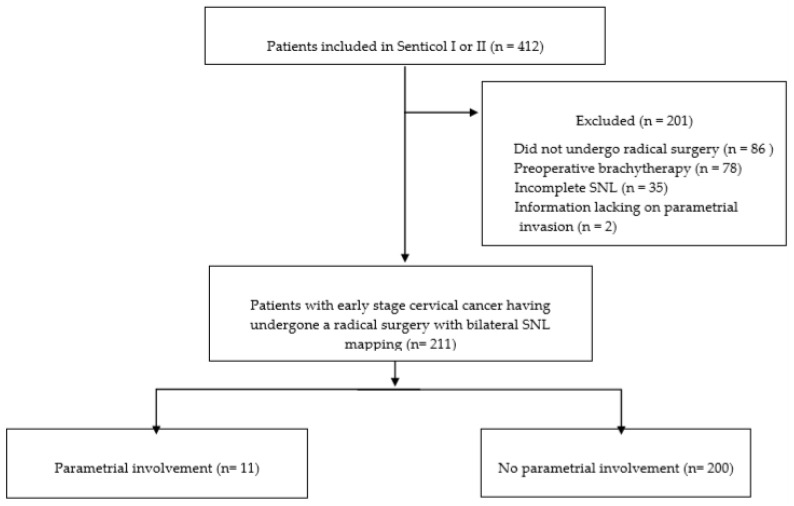
Flow chart.

412 patients were enrolled in 23 French centers from January 2005 to July 2012. Among them, 326 underwent a radical surgery (radical trachelectomy or hysterectomy) Of those enrolled, 115 patients were excluded—78 patients had preoperative brachytherapy, 6 patients had no SLNs detected, 29 patients had SLNs detected on only one side and 2 patients had missing data about parametrial status at final pathologic examination. A total of 211 patients fulfilled the inclusion criteria and were analyzed, as shown in Figure 1.

In the whole population, the mean age was 43.2 years old and the mean BMI (body mass index) was 23.5 kg/m^2^. Patients more frequently underwent a radical hysterectomy (75.8%) and SLN biopsy with additional pelvic lymphadenectomy (69.2%). The most frequent surgical route was laparoscopic (88.7%), followed by laparotomy (8.5%) and robotic (2.8%). On final pathological examination, the most frequent histology was squamous cell carcinoma (67.3%) and the mean tumor size was ten millimeters. In total 182 patients (86.3%) had a negative SLN. Patient characteristics are presented in Table 1.

### 3.2. Likelihood of a Parametrial Involvement

Overall, 11 patients (5.2%) had PI on surgical specimens. Seven patients had unilateral parametrial involvement whereas four patients had bilateral parametrial involvement. 

Patients with PI had a higher mean BMI (23.3 versus 27.1 kg/m^2^ *p* = 0.02), more pathological SLN (54.5% versus 11.5% *p* < 0.001) with more macrometastases (27.3% versus 2.5%, *p* < 0.001). On final pathological exam, patients with PI had larger tumors (28.5mm versus 9 mm, *p* < 0.001), more DSI (17.6 mm versus 4.3 mm *p* < 0.001), LVSI (81.8% versus 31% *p* = 0.001), vaginal invasion (54.5% versus 2.5%, *p* < 0.001) and positive margins (36.4% versus 3% *p* < 0.001). The ideal threshold of DSI predicting PI was 10 mm with a sensibility of 72.73% and a specificity of 81.59%.

After univariate analysis, BMI (odds ratio, OR = 1.1 IC95% = 1.01–1.22 *p* = 0.03), SLN status (*p* < 0.001), tumor size (OR = 18 IC95% = 3.7–86.7 *p* < 0.001), DSI (OR = 14.5 IC95% = 2.9–71.2 *p* < 0.001) and LVSI (OR = 10.1 IC95% = 2.1–47.7 *p* < 0.001) were associated with PI. Only SLN status remained significantly associated with PI after multivariate analysis, especially for macrometastases, as shown in Table 2.

### 3.3. Development of the Model

**Figure 2:**
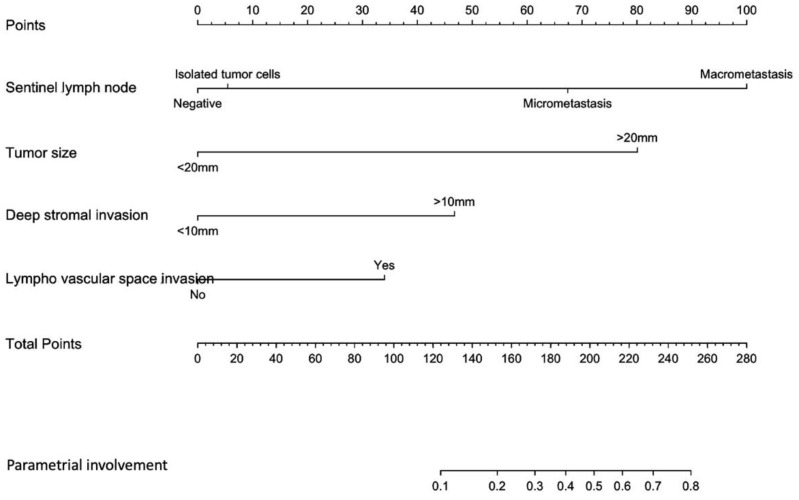
Nomogram predicting the likelihood of a parametrial involvement in patients with early stage cervical cancer. The probability of a parametrial involvement is calculated by drawing a line to the corresponding point on the axis for each of the following variables: tumor size; sentinel lymph node; deep stromal invasion; lympho-vascular space invasion. The points accumulated by the covariates are summed up and correspond to the “total points”. Next, a vertical line is projected from the total points line to the predicted probability bottom scale to obtain the individual probability of a parametrial invasion.

SLN status (ITC, micrometastases, macrometastases), tumor size (<20 mm or ≥20 mm), DSI (<10 mm or ≥10 mm) and presence of LVSI were included in our predictive model, as shown in Figure 2. 

**Figure 3:**
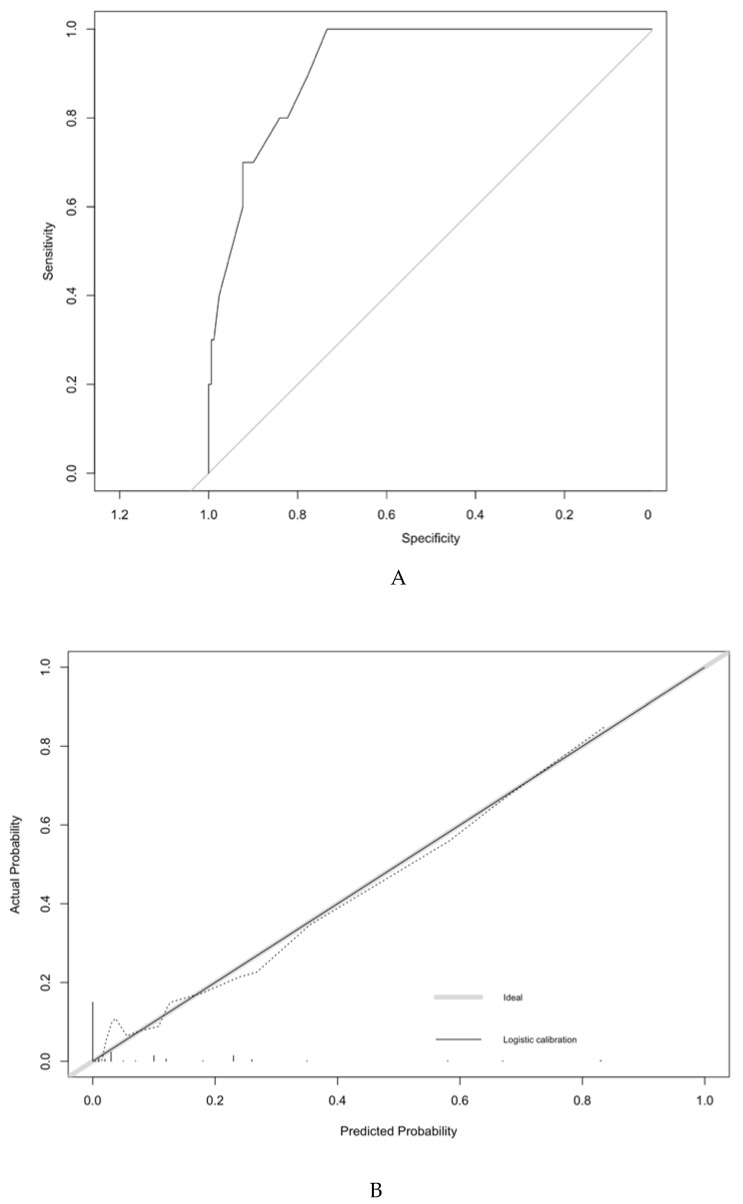
Discrimination and validation of the model predicting the likelihood of a parametrial involvement in patients with early stage cervical cancer. ROC curve of the model. The predictive model had an AUC of 0.92 (IC95% = 0.86–0.98). For the calibration of the model, the horizontal axis represents the predicted probability of a parametrial involvement, and the vertical axis represents the actual probability of parametrial invasion. Perfect prediction would correspond to the 45-degree broken line. The dotted and solid lines indicate the observed (apparent) nomogram performance before and after bootstrapping.

The predictive model had a satisfactory discriminatory power with an AUC of 0.92 (IC95% = 0.86–0.98) before the 200 repetitions of bootstrap sample corrections, as shown in Figure 3. The calibration was also adequate, and no significant difference was noted between the predicted probability obtained from the bootstrap correction and the actual probabilities of a PI (*p* = 1), as shown in Figure 2. The average and maximal differences in predicted and calibrated probabilities were 0.02 and 0.07%, respectively.

The optimal threshold was defined by the Youden index. Patients with a predicted probability < 10% or ≥ 10% presented a probability of a PI of 2.1% and 31.8%, respectively. This threshold had sensitivity, specificity, predictive positive and negative predicted values of 63.6%, 92.5%, 31.8% and 97.8%, respectively.

## 4. Discussion

In this study, we aimed to propose a simple score predicting parametrial involvement in patients with early-stage cervical cancer. This tool could avoid unjustified radical hysterectomy or trachelectomy in patients who would not benefit from one in terms of survival. Simple and readily available variables, such as BMI, tumor size, SLN involvement, LVSI and DSI were integrated in our model. Our score proposes an individual probability of PI. The subgroup of patients with a predicted probability < 10% can be considered as a low-risk group with a probability of PI of 2%.

Initial studies evaluating prognostic factors associated with PI included definitive lymph node status in their analysis [1,17,18]. However, negative SLN can also accurately predict PI [20,29]. In our study 20.7% of patients with a positive SLN had a PI concordant with the 28% found by Strnad et al [30]. In multivariate analysis, a positive SLN was strongly associated with a PI (OR = 16.34 IC95% = 1.33–199.89, *p* = 0.03).

Most variables included in our nomogram, such as pathological tumor size, LVSI and DSI, are not available pre-operatively, but they may be assessed on the conization sample. Even if the negative predictive value of LVSI on conization sample is still debated, data are still lacking concerning its predictive power [31]. Moreover, tumor size can be determined via manual rectovaginal examination, MRI, conization or final pathological analysis, and studies have not yet shown the superiority of one measurement technic. Covens et al., in his large prospective study chose clinical tumor size as a predictive factor, whereas Stegeman et al. used conization sample size, Frumovitz et al. used final pathological size and Yamazaki et al. used MRI size [1,18,19,32]. All found a low risk groups with a probability of PI of 0–1.94%. This size can be closely linked to the size of the conization sample using our 20 mm threshold. A recent meta-analysis showed that ultrasound could also be an alternative to MRI [33]. 

Likewise, LVSI and DSI were included in our score and are not available pre-operatively but present on the conization sample. Indeed, LVSI and DSI are highly prognostic factors [1,17,34]. The optimal threshold of DSI predicting PI was 10 mm, concordant with recent works [1,32,35]. Parametrial invasion spreads through LVSI in up to 52% of the cases, thus explaining the role of this factor [10]. Patients with no LVSI, a tumor diameter < 20 mm and no lymph node involvement have a low incidence of PI of 0.4% [17]. In our cohort, contrary to what has already been described, age or menopausal status was not associated with PI [2,3]. This may be caused by the relatively young age of the patients included in our study [4,36]. 

In the era of personalized medicine, despite many studies addressing the issue of predicting PI, they rarely present the likelihood of PI for each individual patient. Kong et al. proposed a nomogram to pre-operatively predict PI in early-stage cervical cancer [3]. In this study, menopausal status, specific MRI measures (diameter-based ellipsoid tumor volume and the disruption of the cervical stromal ring) and serum squamous cell carcinoma antigen (SCC) tumor markers were included in their nomogram. This score can be difficult to apply to a general population. First, the suggested specific MRI criteria require expert radiologists and would be less reproducible in a non-specialized center. Secondly, using the SCC tumor marker in a population containing non-squamous cell cancers may be controversial. Only 21.3% of the patients in the parametrical invasion group had a squamous cell carcinoma versus 78.7% in the negative group. Lastly, their high rate of PI (21.5%) may raise the question of the extrapolation of this model.

According to current guidelines, the surgical management of early-stage cervical cancer consists in radical hysterectomy and lymph node staging [16]. Pelvic lymphadenectomy is the gold-standard for lymph node staging, although association with SLN biopsy is strongly recommended. However, combined treatment (surgery and adjuvant chemoradiation) should be avoided due to increased morbidity. According to our results, we support the idea that early stage cervical cancer should be managed with a two-step approach [4]. Patients would first undergo a low-invasive surgical assessment consisting in conization and SLN mapping. By using our predictive model, pathologic data obtained from conization specimen, such as tumor size, LVSI, DSI, and the ultrastaging of SLN, would determine low-risk and high-risk patients. Patients with a low risk of PI would undergo a simple hysterectomy or trachelectomy, whereas patients with a high-risk of PI would benefit from a radical surgery. This tool can be used for patients benefiting from a radical hysterectomy or trachelectomy, since both require a similar parametrial resection.

This two-step strategy may present undeniable benefits. First, it would avoid a useless radical surgery in patients with low risk of PI. Indeed, the high negative predictive value (97.8%) of our tool show that this sub-group of patients present a very low risk of PI (2%). Patients would benefit from a simpler surgery with a lower risk of complications. However, it has to be emphasized that radical hysterectomy or trachelectomy may be more difficult after previous SLN dissection, but the indication of radical surgery would be reduced by using this score. In addition, a two-step approach may lead to potential difficulties in terms of organization (two general anesthesia, two operating room slots) and increased costs. This has to be balanced with the benefits of an avoided radical surgery with less blood loss, less transfusion, less complications and, therefore, less increased costs associated with complications [37,38]. Patients could benefit from a same-day discharge conization and SLN mapping, followed by an ambulatory simple hysterectomy or trachelectomy [39]. No over-night stay would be necessary in the low risk group. We are waiting for the results of the prospective trial of Marie Plante et al., SHAPE (NCT01658930), which is an international randomized trial comparing the safety and morbidity of radical hysterectomy and pelvic node dissection with simple hysterectomy and pelvic node dissection in early-stage cervical cancer [40].

Intraoperative SLN status assessment has a variable diagnostic value and frozen sections of SLN are not sufficiently reliable yet, especially for low-volume metastases [21,41,42,43] even if their prognostic value is still a subject of debate [44,45]. Ultrastaging is required to identify missed micrometastases and macrometastases. Moreover, the recommended surgical approach for locally advanced cervical cancer is laparotomy, since the publication of the LACC trial results [46]. For patients with metastatic SLN, a two-step approach would avoid a useless laparotomy and these patients would be referred to concomitant chemo-radiation.

The current study describes a simple tool to identify patients at low risk of PI, but some limitations must be noted. The retrospective analysis of two prospective databases may suffer from some bias. The small number of patients may limit the power of our work, yet studies evaluating PI are often relatively small, since PI is rare [2,3,4,18,19]. Likewise, the small number of events may result in the overfitting of our tool. However, this multicentric study (9 and 23 French centers) is largely generalizable. No external validation was performed for our score. Due to the small percentage of PI (5.2%) in the general population, we could not split the population into a training and validation cohort. An external validation of our score is therefore needed. Likewise, due to the small number of events, most variables included in our score were not significant after multivariate analysis. We decided to include them because of their clinical pertinence.

Several large studies are currently ongoing to assess the morbidity and safety of a step-down surgery for early-stage cervical cancer, such as SHAPE. Likewise, GOG 278 will assess the morbidity and quality of life of non-radical surgery (NCT01649089). ConCerv is a multi-centric international trial, which aims to determine the safety and feasibility of conservative surgery (i.e. hysterectomy or conization with pelvic node dissection and sentinel mapping). The results have recently been presented and the authors underlined that lymph node status assessment is required and optimal pathological criteria for conservative surgery must still be determined. Our simple tool includes SLN status, which may improve the identification of these patients eligible for conservative surgery.

## 5. Conclusions 

In conclusion, we developed a nomogram based on five clinical and pathological characteristics to predict PI with a high concordance probability. This simple and original tool could be used to determine patients with a low risk of PI and could be eligible for a less radical surgery in a two-step approach. 

## Figures and Tables

**Table 1:** Patient characteristics in the total population, group with and group without parametrial involvement in early stage cervical cancer.

Predictive Variable	Total Population *n* = 211		Group without Parametrial Invasion *n* = 200		Group with Parametrial Invasion *n* = 11		*p*
	*n* or Mean ± SD	(%) or (Range)	*n* or Mean ± SD	(%) or (Range)	*n* or Mean ± SD	(%) or (Range)	
Age (years)							
Mean ± SD	43.2 ± 11.6	(22–85)	43.1 ± 11.6	(22–85)	45.4 ± 12.4	(31–77)	0.52
<50	165	78.2	156	78	9	81.8	0.42
50–70	39	18.5	38	19	1	9.1	
>70	7	3.3	6	3	1	9.1	
BMI (kg/m^2^)							
Mean ± SD	23.5 ± 5.1	(14.6–42.2)	23.3 ± 4.9	(14.6–41.4)	27.1 ± 7.8	(18–42.2)	0.02
<18.5	18	8.5	17	8.5	1	9.1	0.43
18.5–25	139	65.9	133	66.5	6	54.5	
<25–30	29	13.7	28	14.0	1	9.1	
>30	25	11.8	22	11.0	3	27.3	
Parity							
0	50	23.7	48	24.0	2	18.2	0.66
≥1	161	76.3	152	76.0	9	81.8	
Menopausal status							
Yes	52	24.6	49	24.5	3	27.3	0.83
No	159	75.4	151	75.5	8	72.7	
Clinical FIGO stage							
IA1 with emboli-IA2	24	11.5	24	12.1	0	0.0	0.63
IB1	115	55.0	108	54.5	7	63.6	
IB2	67	32.1	63	31.8	4	36.4	
IIA1	3	1.4	3	1.5	0	0.0	
Not specified	2		2				
Histology							
Squamous cell carcinoma	142	67.6	132	67.9	10	90.9	0.23
Adenocarcinoma	61	29.0	60	29.6	1	9.1	
Other type	7	3.3	7	2.6	0	0.0	
Not specified	1		1				
Grade of differentiation							
G1	65	43.3	63	44.7	2	22.2	0.41
G2	58	38.7	53	37.6	5	55.6	
G3	27	18.0	25	17.7	2	22.2	
Not specified	61		59		2	2	
Type of surgery							
Radical Hysterectomy	160	75.8	150	75.0	10	90.9	0.23
Radical Trachelectomy	51	24.2	50	25.0	1	9.1	
Type of Lymph node staging							
SLN biopsy alone	65	30.8	64	32.0	1	9.1	0.11
SLN biopsy + Pelvic lymphadenectomy	146	69.2	136	68.0	10	90.9	
Node status							
Patients with positive SLN							
Yes	29	13.7	23	11.5	6	54.5	<0.0001
No	182	86.3	177	88.5	5	45.5	
SLN status							
Macrometastasis	8	3.8	5	2.5	3	27.3	<0.0001
Micrometastasis	10	4.7	9	4.5	1	9.1	
ITC	11	5.2	9	4.5	2	18.2	
Negative	182	86.3	177	88.5	5	45.5	
Final pathological exam							
Tumor size							
Mean (mm) ± SD	10 ± 11.9	(0–60)	9 ± 11.1	(0–60)	28.5 ± 10.9	(15–50)	<0.0001
<20 mm	158	76.7	156	80	2	18.2	<0.0001
≥20 mm	48	23.3	39	20	9	81.8	
Not specified	5		5				
Deep stromal invasion							
Mean (mm) ± SD	5.6 ± 7.7	(0–40)	4.3 ± 7.8	(0–40)	17.6 ± 8.3	(6–30)	<0.0001
<10 mm	136	75.1	134	78.4	2	20.0	<0.0001
≥10 mm	45	24.9	37	21.6	8	80.0	
Not specified	30		29		1		
Lympho-vascular space invasion							
Yes	71	33.6	62	31.0	9	81.8	0.001
No	140	66.4	138	69.0	2	18.2	
Vaginal invasion							
Yes	11	5.3	5	2.5	6	54.5	<0.0001
No	198	94.7	193	97.5	5	45.5	
Not specified	2		2				
Positive margin							
Yes	10	4.8	6	3.0	4	36.4	<0.0001
No	199	95.2	192	97.0	7	63.6	
Not specified	2		2				

BMI, body mass index; SLN, sentinel lymph node; ITC, isolated tumor cells, SD standard deviation.

**Table 2:** Univariate and multivariate analysis of predictive factors associated with parametrial involvement.

	Univariate			Multivariate		
Variable	Odds Ratio	IC 95%	*p*	ORa	IC 95%	*p*
Body mass index (kg/m^2^)						
	1.1	1.01–1.22	0.03	1.11	0.98–1.27	0.11
Sentinel lymph node status						
Negative	1			1		
ITC	3.93	0.41–37.28	0.23	1.63	0.13–19.65	0.7
Micrometastasis	7.86	1.34–46.24	0.02	9.91	0.53–183.19	0.12
Macrometastasis	21.24	3.94–114.52	<0.001	16.34	1.33–199.89	0.03
Tumor size						
<20 mm	1			1		
≥20 mm	18	3.74–86.68	<0.001	6.55	0.81–53.31	0.08
Deep stromal invasion						
<10 mm	1			1		
≥10 mm	14.49	2.95–71.16	<0.001	5.55	0.49–63.4	0.17
Presence of lympho-vascular space involvement						
No	1			1		
Yes	10.02	2.10–47.72	<0.001	2.25	0.33–15.23	0.41
